# Supplementary material for: Uncertainty quantification and sensitivity analysis of COVID-19 exit strategies in an individual-based transmission model
Source: PLoS Comput Biol. 2021 Sep 17;17(9):e1009355. doi: 10.1371/journal.pcbi.1009355 (PMC8480746; doi:10.1371/journal.pcbi.1009355)
Supplement: S1 Table — Overview of the values of the parameters in the SEIR model. In the last column we indicate the respective parameter in the computational model (or which computational parameter is affected). (PDF) [file pcbi.1009355.s001.pdf]

S1 Table

Overview of parameter values used in the analysis. Overview of the values of the parameters in the SEIR model. In the last column we indicate the respective parameter in the computational model (or which computational parameter is affected).

Parameter	Description	Value(s)	Computational model parameter
ν_E	average incubation time	4.6 days	`exposed_time`
α_E	shape of Weibull distribution for incubation time	20 (considered as uncertain in parts of the analysis)	`shape_exposed_time`
ν_I	average duration of infectiousness	5 days (considered as uncertain in parts of the analysis)	`avg_duration _infectiousness`
α_I	shape of the Weibull distribution for duration of infectiousness	1	`infected_time`
N	total population size	10^6	`n_agent`
K	number of superclusters	20	`n_supercluster`
J	number of cluster per supercluster	1000	`n_cluster`
σ	standard deviation of cluster-level sampling weights for the multinomial distribution of clusters population sized	0.95	`cluster_size_sd`
β	overall contact rate	0.5 (considered as uncertain in parts of the analysis)	`contact_rate`
α	shape and rate of Γ distribution for variations in individual relative contact rates (relative to β)	3.4	`contact_shape`
ϑ	level of assortative mixing	0.26	`contact_assort`
θ_{SC}, θ	weights for transmission coming from superclusters and the general population, respectively; the derived weight for transmission coming within cluster is $1 - \theta_{SC} - \theta$	0.05, 0.05	`population_mixing`, `supercluster _mixing`

μ	relative level to which transmission is reduced on average in supercluster k	(considered as uncertain in parts of the analysis)	`intervention _effect`
τ	size of the Beta distribution for inter-individual variation in the effect of contact-related interventions	∞ (i.e. no variation) (considered as uncertain in parts of the analysis)	`intervention _effect_var`
φ	multiplier for the contribution and exposure of a supercluster to population-level transmission in case of isolation	0.5 in case of isolation of the supercluster as part of the phased opening strategy, 1 otherwise	`sc_isolation _effect`
	total running time in days	550	`runtime`
	standard deviation of the softmax-transformed cluster-level sampling weights for the multinomial distribution of the expected number of clusters per supercluster	0	`supercluster _size_sd`
	external force of infection assumed to be continuously present over time	50/(365 · `n_agent`) for FC and CT; 0 for IL and PO	`efoi`
	initial number of infected cases	50	`infection_init`
	scalar value for cumulative incidence of cases after which interventions are started	9500	`inc_cum_cond`
