# Supplementary material for: Uncertainty quantification and sensitivity analysis of COVID-19 exit strategies in an individual-based transmission model
Source: PLoS Comput Biol. 2021 Sep 17;17(9):e1009355. doi: 10.1371/journal.pcbi.1009355 (PMC8480746; doi:10.1371/journal.pcbi.1009355)
Supplement: S1 Text — (PDF) [file pcbi.1009355.s002.pdf]

S1 Text

Technical model description of virsim. Here we provide a technical description of the geographically stratified SEIR (Susceptible - Exposed - Infectious - Recovered) model used to predict the transmission dynamics of COVID-19 in a country or part of a country. The model has been developed by de Vlas & Coffeng [1] and its source code - in the form of the R package virsim - is available online [2] under the CC BY-NC-ND 4.0 license. S1 Table in the Supporting information provides a complete overview of the parameter values used in the main analysis.

We describe the dynamics of COVID-19 transmission using a standard SEIR model for a closed population of size N, ignoring births and deaths. In terms of ordinary differential equations this can be described as:

$$\begin{aligned}
\frac{d}{dt}S(t) &= -\lambda S \ , \\
\frac{d}{dt}E(t) &= \lambda S - \rho E \ , \\
\frac{d}{dt}I(t) &= \rho E - \gamma I \ , \\
\frac{d}{dt}R(t) &= \gamma I \ ,
\end{aligned}$$

with

$$\lambda = \beta \frac{I}{N} \qquad \text{and} \qquad N = S + E + I + R \ .$$

Here, λ is the force of infection, β is the average contact rate in the population (including the average probability that transmission occurs during an average contact), ρ is the inverse of the average incubation time, and γ is the inverse of the average duration of infectiousness, assuming exponentially distributed sojourn times. To relax assumptions about exponentially distributed durations and to capture various other heterogeneities (more details below), in virsim the SEIR model is implemented in an individual-based framework in discrete time (one-day time steps). We assume that the durations of compartments E and I each follow a Weibull distribution with mean ν_E and ν_I and shape α_E and α_I. Infection events (transitions from S to E) are assumed to follow an exponential distribution, with the probability of an individual being infected on day t defined as:

$$p_t\left(S \rightarrow E\right) = 1 - \exp\left(-\Delta t \lambda_t\right) \ .$$

To capture differential mixing of individuals in the same community (e.g. a town, ward or village) and administrative unit (e.g. a province), we distribute all N individuals over K superclusters that each consist of N_k individuals. Within each supercluster, we further subdivide the N_k individuals over J_k clusters of N_{jk} individuals. We allow for variation in cluster size N_{jk} by distributing the population over all $\sum_{k=1}^{K} J_k$ clusters using a multinomial distribution with cluster-specific probability weights drawn from a log-normal distribution

with mean 0 and standard deviation σ. We assume that each supercluster contains the same number of clusters (i.e. unrelated to individual cluster sizes), so $J_k = J$, for all $k = 1, ..., K$.

To capture heterogeneity in contact rates of individuals and potential assortative mixing of individuals with similar transmission-related behavior, we assign each individual a life-long weight w_{ijk} which represents the individual's contact rate relative to the population average contact rate β. We allow relative contact rates to vary between individuals according to a Γ distribution with equal shape and rate α such that the average relative contact rate is one. Relative contact rates capture inter-individual variation resulting from both contact frequency and the probability of transmission per contact.

Assortative mixing (i.e. differential mixing of individuals with more similar contact rates) is captured by drawing and assigning individual relative contact rates w_{ijk} as follows. First, after assigning individuals to clusters, for each individual i we draw a random value x_i from the unit normal distribution $\mathcal{N}(0, 1)$. Likewise, for each cluster j we draw a random value $x_j \sim \mathcal{N}(0, 1)$. For each individual i in each cluster j we then add up $x_{ij} = (1 - \vartheta)x_i + \vartheta x_j$, where ϑ is a parameter between 0 and 1 representing the level of assortative mixing. We then determine the rank of each individual in the entire population based on their value x_{ij}. Next, we draw N values of $w_{ijk} \sim \Gamma(\alpha, \alpha)$ and order these (ascending or descending order does not matter). Then finally, we assign each individual the nth of the ordered values of w_{ijk}, where n is the individual's rank in terms of x_{ij}. If $\vartheta = 0$, there is no assortative mixing and cluster-level average contact rates are all 1 ($\pm$ Monte Carlo sampling variation). If $\vartheta = 1$, there is maximum assortative mixing such that individuals' relative contact rates w_{ijk} within a cluster are extremely similar and the cluster-level average contact rates follow a Γ distribution $\Gamma(\alpha, \alpha)$.

To account for the impact of heterogeneity in individual contact rates, geographical mixing patterns, and potential assortative mixing on transmission, we define the force of infection λ_{ijk} acting on a susceptible individual i in cluster j in supercluster k as:

$$\begin{aligned}
\lambda_{ijk} &= \varepsilon_{ik} w_{ijk} \beta \cdot \\
&\left((1 - \theta_{SC} - \theta) \frac{1}{N_{jk}} \sum_{p=1}^{N_{jk}} \Lambda_{pjk} + \theta_{SC} \frac{1}{N_k} \sum_{q=1}^{J_k} \sum_{p=1}^{N_{qk}} \Lambda_{pqk} + \varphi_k \theta \frac{1}{N} \sum_{r=1}^{K} \varphi_r \sum_{q=1}^{J_r} \sum_{p=1}^{N_q r} \Lambda_{pqr} \right) , \\
\Lambda_{ijk} &= \varepsilon_{ik} w_{ijk} \mathrm{I}(I_{ijk}) , \\
\varepsilon_{ik}^2 &\sim \mathrm{Beta}\left(\mu_k \tau, (1 - \mu_k)\tau\right) .
\end{aligned}$$

Here, $\mathrm{I}(I_{ijk})$ is an indicator function that returns 1 if individual i in cluster j in supercluster k is in the compartment I (i.e. infectious), and 0 otherwise. Interventions aimed at reducing contact rates (e.g. social distancing) are assumed to be implemented at the level of superclusters; their effect ε_{ik} on contact rates may vary between individuals, with its square following a Beta distribution with mean μ and size τ (i.e. the sum of the distribution's shape parameters). In case

the reduction of contact rates is the same across and within all superclusters ($\mu_k = \mu \; \forall k = 1, ..., K$ and $\tau \to \infty$, such that $\varepsilon_{ik} = \sqrt{\mu}$), the quantity $\varepsilon_{ik}^2 = \mu$ represents the reduction in the overall contact rate β (i.e. the quantity reported in the main manuscript). However, to capture the effect of implementing control in only part of the population (i.e. when $\mu_k \neq \mu$), we define μ_k at the supercluster level. Changes in μ_k over time can be specified per supercluster, allowing the simulation of a geographically heterogeneous intervention. In case μ_k changes over time, individual reductions ε_{ik} in contact rates are assumed to change proportionally to $\sqrt{\mu_k}$, and to be stable over time otherwise (reflecting the individual's inclination to adhere to control). Although not described above, the model also includes a mechanism that allows the user to specify which (random) fraction of the population will take up the intervention at each time point (default value 100%). For individuals who do not take up the intervention, we assume $\varepsilon_{ik} = 1$.

Differential mixing of populations in clusters and superclusters is captured by mixing weights for population-level transmission (θ), supercluster-level transmission (θ_{SC}), and cluster-level transmission ($1 - \theta_{SC} - \theta$). Isolation of a supercluster for control of transmission is simulated by multiplying a supercluster's contribution and exposure to population-wide transmission by φ (range 0–1).

In absence of variation in individual contact rates (i.e. $w_{ijk} = 1$) and in case of homogeneous mixing of the population ($\theta = 1$, and $\theta_{SC} = 0$) and uniform implementation of (and adherence to) control measures (i.e. $\varepsilon = \sqrt{\mu}$), the above equation for λ_{ijk} can be reduced to the original formulation of the force of infection in a simple SEIR model:

$$\lambda = \mu\beta \frac{1}{N} \sum_{k=1}^{K} \sum_{j=1}^{J_k} \sum_{i=1}^{N_i jk} \mathrm{I}(I_{ijk}) = \mu\beta \frac{1}{N} \ .$$

References

[1] De Vlas S, Coffeng LE. Achieving herd immunity against COVID-19 at the country level by the exit strategy of a phased lift of control. Sci Rep. 2021;11(4445). doi:10.1038/s41598-021-83492-7.

[2] Coffeng LE. virsim; 2020. `https://gitlab.com/luccoffeng/virsim/-/tree/v1.0.5`.
