# Supplementary material for: Uncertainty quantification and sensitivity analysis of COVID-19 exit strategies in an individual-based transmission model
Source: PLoS Comput Biol. 2021 Sep 17;17(9):e1009355. doi: 10.1371/journal.pcbi.1009355 (PMC8480746; doi:10.1371/journal.pcbi.1009355)
Supplement: S2 Text — (PDF) [file pcbi.1009355.s003.pdf]

S2 Text

Numerical implementation of the strategies. All strategies share the initial sequence of interventions which represent

- the spreading of the virus among the population
- the beginning of the general lockdown
- the imposition of a stricter lockdown
- the reopening of the schools
- the application of the proposed exit strategy.

The start of the epidemic is simulated by randomly seeding 10 infections. When the amount of infected agents reaches a certain value (model parameter `inc_cum_cond`), a general lockdown is established. One week later a stricter lockdown is imposed (roughly mimicking the history of the Dutch lockdown in spring 2020) until schools are reopened 53 days later. Thirty days later the selected strategy is applied.

From a numerical point of view, the interventions are described by the parameters `intervention_t` (a vector of time points at which interventions change) and `intervention_effect` (a vector of the time-specific effect of interventions - i.e. a multiplier - for the average transmission rate in the population). These two parameters have to be defined for all strategies and additional strategy-dependent variables might be required (see Methods and model section in the main text).

The initial sequence of interventions shared by all strategies is implemented as follows:

- `intervention_t` = cumsum([0, 10, 7, 53, 30, α]) where cumsum indicates that the components of the resulting vector are the cumulative sums and the second component is dynamically adapted such that the lockdown starts when there are `inc_cum_cond` infected individuals;
- `intervention_effect` = [1, 0.3, 0.15, 0.25, β].

We use the Greek letters α and β to indicate either single values or sequences of values that define the strategies. More details about their specific definition in the cases considered follow below. α is necessary only in case of Intermittent Lockdown or Phased Opening and can be omitted in Flattening the Curve or in Contact Tracing.

Flattening the Curve. For this strategy it is not necessary to extend further the time series of interventions, i.e. `intervention_t` = cumsum([0, 10, 7, 53, 30]), and β is modelled by the uncertain parameter `int_effect` such that `intervention_effect` = [1, 0.3, 0.15, 0.25, `int_effect`]. We assume that the population uptake is constant in time, hence `uptake` is kept unchanged for the

whole simulation, i.e. `intervention_uptake` = rep(`uptake`, len(`intervention_t`)) where `uptake` is repeated as many times as the length of `intervention_t`

Contact Tracing. Also in this strategy we do not require α and the time series of interventions is given by `intervention_t` = cumsum([0, 10, 7, 53, 30]). Since the restrictions imposed by CT are determined by the variables `trace_rate_I`, `trace_prob_E` and `trace_contact_reduction` and not by `intervention_effect`, we set $\beta = 1$, i.e. `intervention_effect` = [1, 0.3, 0.15, 0.25, 1]. We assume that the CT measures are not active before the actual start of the strategy, hence we have `trace_prob_E` = [0, 0, 0, 0, `trace_E`], `trace_rate_I` = [0, 0, 0, 0, `trace_I`] and `trace_contact_reduction` = [0, 0, 0, 0, `trace_cr`] where `trace_E`, `trace_I` and `trace_cr` are the uncertain parameters discussed in the main text.

Intermittent Lockdown. The interchange between lockdowns and lifts is modelled by `intervention_t` and `intervention_effect`. In particular, α consists in a phase of lift after the opening of the schools - necessary to let the virus spread again among the population - and the repetition of the length of the lockdown and subsequent lift. The duration of the very first lift phase after the general lockdown is largely determined by the initial state of the epidemic at the start of the strategy, thus it is not useful to consider it as uncertain and we set it to 25 days for our analyses. The final time series of the interventions reads `intervention_t` = cumsum([0, 10, 7, 53, 30, 25, rep((`lockdown_length`, `lift_length`),10)]) where (`lockdown_length`, `lift_length`) is being repeated 10 times.

Similarly β consists in the alternation of the effect on the average contact rate due to the lockdown or the opening, i.e. `intervention_effect` = [1, 0.3, 0.15, 0.25, rep((1, `lockdown_effect`),11)] where (1, `lockdown_effect`) is repeated 11 times to match the length of `intervention_t`. As for Flattening the Curve, `uptake` is assumed not to vary during the simulation, i.e. `intervention_uptake` = rep(`uptake`, len(`intervention_t`)).

Phased Opening. The phased lift plan is generated by the function `gen_phased_lift` and does not require extra ad-hoc numerical coding. This function includes several input parameters, of which only some have been considered here as uncertain, i.e. `pl_intervention_effect_hi`, `intervention_lift_interval` and `uptake`. We refer the reader to the documentation of the virsim model provided in its GitLab repository [1] (script `gen_intervention.r` in the `R` folder) for a detailed description of the `gen_phased_lift` function, its inputs and its outputs.

References

[1] Coffeng LE. virsim; 2020. https://gitlab.com/luccoffeng/virsim/-/tree/v1.0.5.
