# Supplementary material for: Uncertainty quantification and sensitivity analysis of COVID-19 exit strategies in an individual-based transmission model
Source: PLoS Comput Biol. 2021 Sep 17;17(9):e1009355. doi: 10.1371/journal.pcbi.1009355 (PMC8480746; doi:10.1371/journal.pcbi.1009355)
Supplement: S3 Text — (PDF) [file pcbi.1009355.s004.pdf]

S3 Text

Theory of Sobol indices. Here we will briefly outline the theory and the algorithm by which we compute the Sobol indices. For a more detailed description we refer to [1]. Let $Y = \mathcal{M}(X_1, \cdots, X_p)$ be the output of a computational model, with p independent inputs such that the joint pdf $p(\mathbf{X})$ is given by $\prod_{i=1}^{p} p(X_i)$. Then, the output variance, conditional on a *fixed* value $X_j = \widetilde{X}_j$ (where $j \in \{1, \cdots, p\}$), is written as

$$\mathbb{V}\mathrm{ar}_{\sim j}\left[Y \mid X_j = \widetilde{X}_j\right] = \\ \int_{\Omega_{\sim j}} \mathcal{M}^2\left(X_1, \cdots, \widetilde{X}_j, \cdots, X_p\right) \prod_{\substack{i=1 \\ i\neq j}}^{p} p\left(X_i\right) \mathrm{d}X_i - \mathbb{E}^2_{\sim j}\left[Y \mid X_j = \widetilde{X}_j\right]. \quad (1)$$

Note that $\Omega_{\sim j}$ indicates integration over the support of all inputs pdfs, except that of $p(X_j)$. Similarly, Ω_j denotes integration over only the support of $p(X_j)$, and we will use Ω to denote integration over the entire stochastic input domain. Also, similar to the main text, we write $\mathbb{E}_i$ and $\mathbb{V}\mathrm{ar}_i$ for expectation and variance over X_i only, $\mathbb{E}_{\sim i}$ and $\mathbb{V}\mathrm{ar}_{\sim i}$ for expectation and variance over all $X_1, X_2, ..., X_p$ except X_i, and $\mathbb{E}$ and $\mathbb{V}\mathrm{ar}$ for expectation and variance over all $X_1, X_2, ..., X_p$.

Expression (1) allows one to gauge the effect that keeping one input fixed has on the output variance. Sobol indices, defined later on, are global sensitivity measures however, so we wish to eliminate the dependence on the specific, local value $\widetilde{X}_j$. We therefore integrate the conditional variance (1) over all $\widetilde{X}_j$ values, i.e.

$$\mathbb{E}_j\left[\mathbb{V}\mathrm{ar}_{\sim j}\left[Y \mid X_j\right]\right] = \\ \int_{\Omega_j}\left[\int_{\Omega_{\sim j}} \mathcal{M}^2\left(X_1, \cdots, \widetilde{X}_j, \cdots, X_p\right) \prod_{\substack{i=1 \\ i\neq j}}^{p} p\left(X_i\right) \mathrm{d}X_i\right] p\left(\widetilde{X}_j\right) \mathrm{d}\widetilde{X}_j \\ - \int_{\Omega_j} \mathbb{E}^2_{\sim j}\left[Y \mid X_j = \widetilde{X}_j\right] p\left(\widetilde{X}_j\right) \mathrm{d}\widetilde{X}_j \\ = \int_{\Omega} \mathcal{M}^2\left(X_1, \cdots, \widetilde{X}_j, \cdots, X_p\right) \prod_{i=1}^{p} p\left(X_i\right) \mathrm{d}X_i - \int_{\Omega_j} \mathbb{E}^2_{\sim j}\left[Y \mid X_j = \widetilde{X}_j\right] p\left(\widetilde{X}_j\right) \mathrm{d}\widetilde{X}_j.$$

Again, this is the expected value of the variance of Y, while keeping one input (X_j) fixed. Hence, if we subtract this from the total variance of Y, we get the contribution to the variance due to X_j alone:

$$\mathbb{V}\mathrm{ar}\left[Y\right] - \mathbb{E}_j\left[\mathbb{V}\mathrm{ar}_{\sim j}\left[Y \mid X_j\right]\right] = \int_{\Omega_j} \mathbb{E}^2_{\sim j}\left[Y \mid X_j = \widetilde{X}_j\right] p\left(\widetilde{X}_j\right) \mathrm{d}\widetilde{X}_j - \mathbb{E}^2\left[Y\right], \quad (2)$$

where $\mathbb{V}\mathrm{ar}[Y] := \mathbb{E}[Y^2] - \mathbb{E}^2[Y]$. We can write the above difference between the variance and the expectation of the conditional variance, as the variance of the

conditional expectation:

$$\begin{aligned}\mathbb{V}\mathrm{ar}_j\left[\mathbb{E}_{\sim j}\left[Y \mid X_j\right]\right] &:= \\ \int_{\Omega_j}\left(\mathbb{E}_{\sim j}\left[Y \mid X_j=\widetilde{X}_j\right]-\mathbb{E}_j\left[\mathbb{E}_{\sim j}\left[Y \mid X_j=\widetilde{X}_j\right]\right]\right)^2 p(\widetilde{X}_i)\mathrm{d}\widetilde{X}_j &= \\ \int_{\Omega_j}\mathbb{E}_{\sim j}^2\left[Y \mid X_j=\widetilde{X_j}\right] p\left(\widetilde{X}_j\right)\mathrm{d}\widetilde{X}_j-\mathbb{E}^2\left[Y\right],\end{aligned}$$

which equals (2). The first-order Sobol indices S_j are defined as the ratio of the variance due to X_j alone, i.e. (3), over the total variance. This gives

$$S_j := \frac{\mathbb{V}\mathrm{ar}_j\left[\mathbb{E}_{\sim j}\left[Y \mid X_j\right]\right]}{\mathbb{V}\mathrm{ar}\left[Y\right]}. \tag{3}$$

Note that this is the expression found in the main text. The appearance of the term $\mathbb{V}\mathrm{ar}_j\left[\mathbb{E}_{\sim j}\left[Y \mid X_j\right]\right]$ might suggest an implementation involving a double loop, where the inner loop computes $\mathbb{E}_{\sim j}^2\left[Y \mid X_j=\widetilde{X}_j\right]$, and the outer loop computes the outer integral. If we denote M as the number of MC samples, the involved cost would be M^2. However, Saltelli [1] developed an algorithm, through which the cost of computing the first-order indices is reduced to $M\left(p+1\right)$. Briefly, several input matrices are constructed, of which the first two are filled with independent draws from $p\left(\mathbf{X}\right)$:

$$A=\begin{bmatrix} x_{11} & \cdots & x_{1p} \\ \vdots & \ddots & \vdots \\ x_{M1} & \cdots & x_{Mp}\end{bmatrix} \quad \text{and} \quad B=\begin{bmatrix} x'_{11} & \cdots & x'_{1p} \\ \vdots & \ddots & \vdots \\ x'_{M1} & \cdots & x'_{Mp}\end{bmatrix} \tag{4}$$

We can think of A as the 'sample' matrix, and B as the 'resample' matrix. Next, p matrices are constructed, where the j-th column of B is replaced by the j-th column vector of A:

$$B_j=\begin{bmatrix} x'_{11} & \cdots & x_{1j} & \cdots & x'_{1p} \\ \vdots & & \vdots & & \vdots \\ x'_{M1} & \cdots & x_{Mj} & \cdots & x'_{Mp}\end{bmatrix}, \quad j=1,\cdots,p. \tag{5}$$

We can now approximate the first-order Sobol indices as follows:

$$S_j := \frac{\mathbb{V}\mathrm{ar}_j\left[\mathbb{E}_{\sim j}\left[Y \mid X_j\right]\right]}{\mathbb{V}\mathrm{ar}\left[Y\right]} = \frac{\int_{\Omega_j}\mathbb{E}_{\sim j}^2\left[Y \mid X_j=\widetilde{X_j}\right] p\left(\widetilde{X}_j\right)\mathrm{d}\widetilde{X}_j-\mathbb{E}^2\left[Y\right]}{\mathbb{V}\mathrm{ar}[Y]} \approx \frac{\widehat{U}_j-\mathbb{E}^2[Y]}{\mathbb{V}\mathrm{ar}[Y]}, \tag{6}$$

where

$$\widehat{U}_j=\frac{1}{M-1}\sum_{r=1}^{M}\mathcal{M}(x_{r1},\cdots,x_{rp})\mathcal{M}(x'_{r1},\cdots,x_{rj},\cdots,x'_{rp}). \tag{7}$$

This single-loop MC approximation corresponds to the integral $\int_{\Omega_j} \mathbb{E}^2_{\sim j}[Y \mid X_j = \widetilde{X}_j] p(\widetilde{X}_j) \mathrm{d}\widetilde{X}_j$, see [1] for details.

We can estimate the unconditional mean and variance in (6) by evaluating the model $\mathcal{M}$ on the M samples of A. To estimate the U_j, we can see from (7) that this requires evaluating the model on the rows of B_j, $j = 1, \cdots, p$, where all inputs except X_j are resampled. This brings the total cost up to $M(p+1)$. If one wishes to also compute the total-order indices, the cost will be increased to $M(p+2)$, see [1] for details.

References

[1] Saltelli A. Making best use of model evaluations to compute sensitivity indices. Comput Phys Commun. 2002;145:280–297.
