# Supplementary figures and images for: Uncertainty quantification and sensitivity analysis of COVID-19 exit strategies in an individual-based transmission model
Source: PLoS Comput Biol. 2021 Sep 17;17(9):e1009355. doi: 10.1371/journal.pcbi.1009355 (PMC8480746; doi:10.1371/journal.pcbi.1009355)

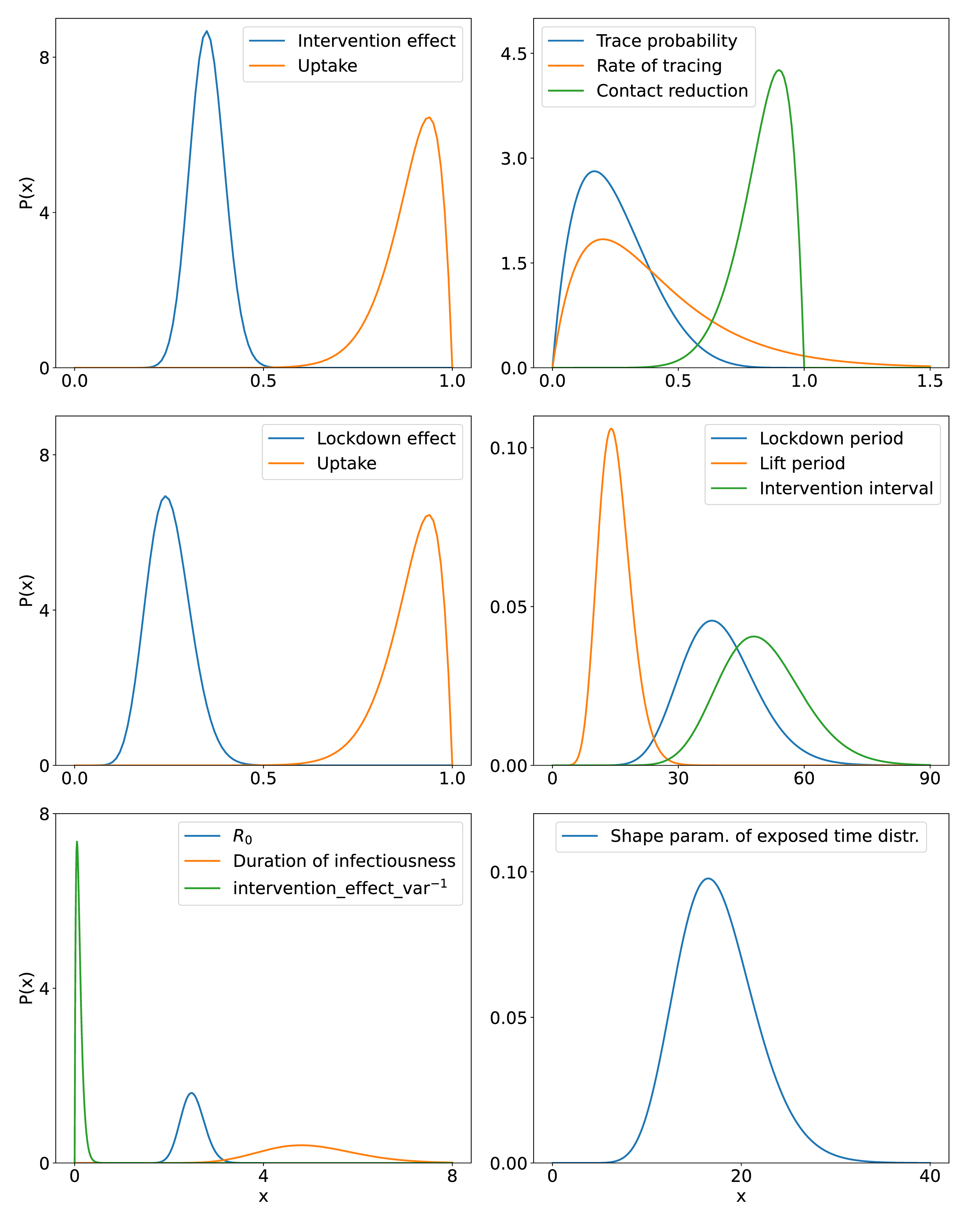

Supplement: S1 Fig — Distributions for the uncertain input parameters in case of Flattening the Curve (top left), Contact Tracing (top right), Intermittent Lockdown and Phased Opening (middle), and for the biology-related parameters (bottom). (TIF) [file pcbi.1009355.s005.tif]

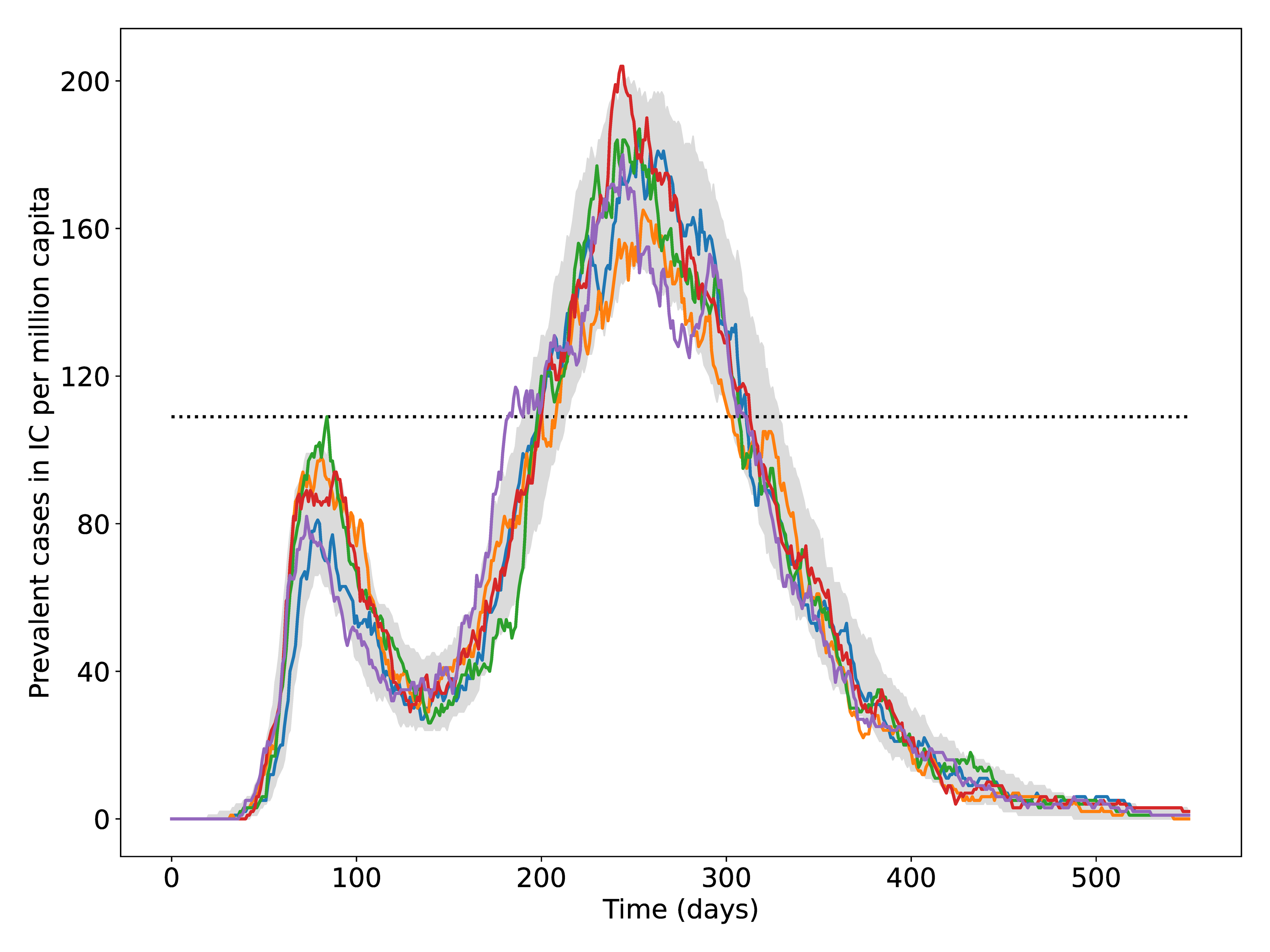

Supplement: S2 Fig — Example of strategy outcome variability due to intrinsic stochasticity of the virsim model. The shaded gray area denotes the interval between the 5th and the 95th percentiles out of 100 realizations of the FC strategy with same policy- and non-policy-related parameters but different seed. A few individual realizations are shown in colored lines. (TIF) [file pcbi.1009355.s006.tif]

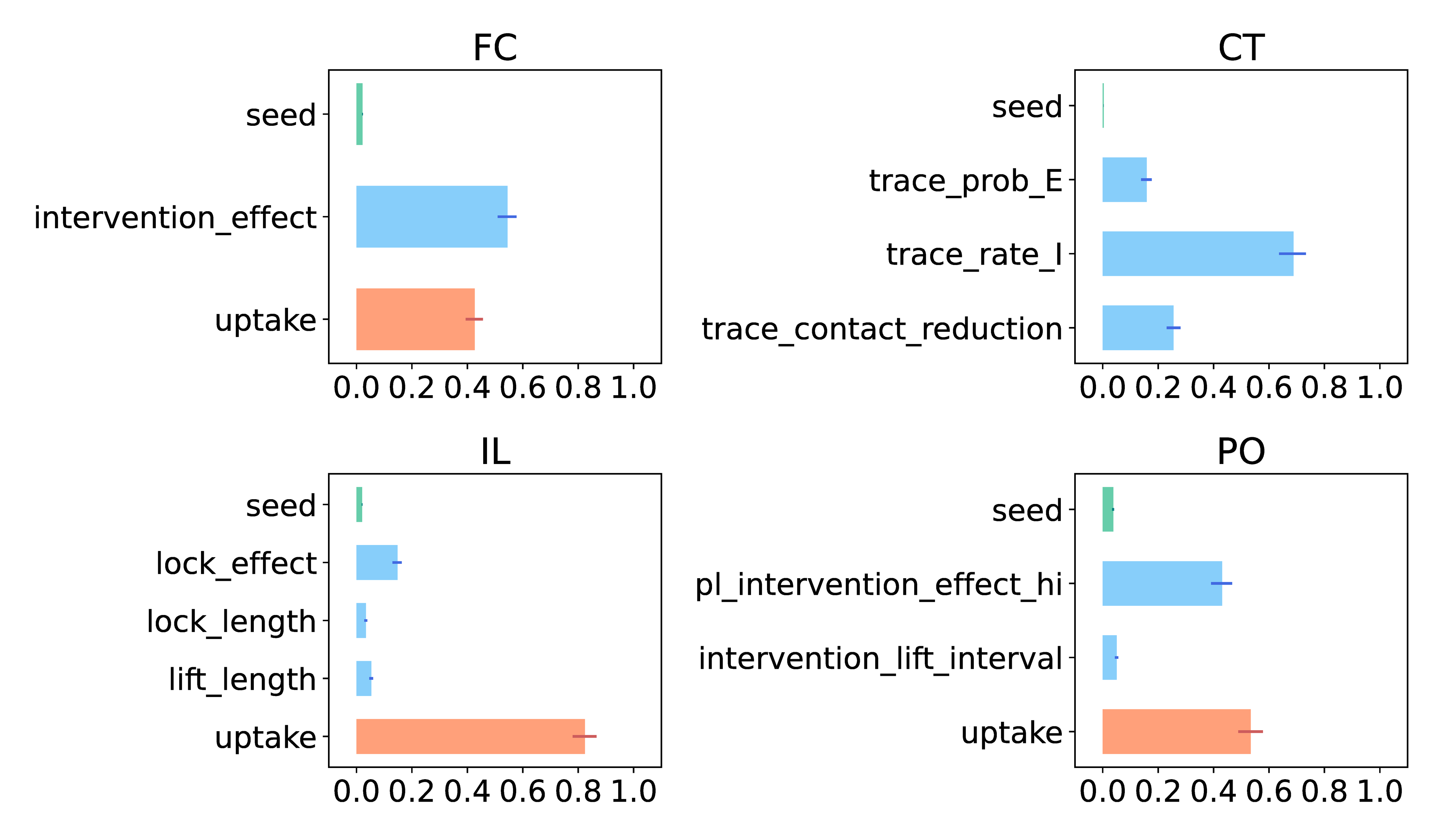

Supplement: S3 Fig — Total Sobol indices of the first QoI (the maximum number of patients in IC). The length of the bars indicate the mean values, while the thinner lines display the 95% confidence interval. We color in orange the uptake parameter, and in green seed. (TIF) [file pcbi.1009355.s007.tif]

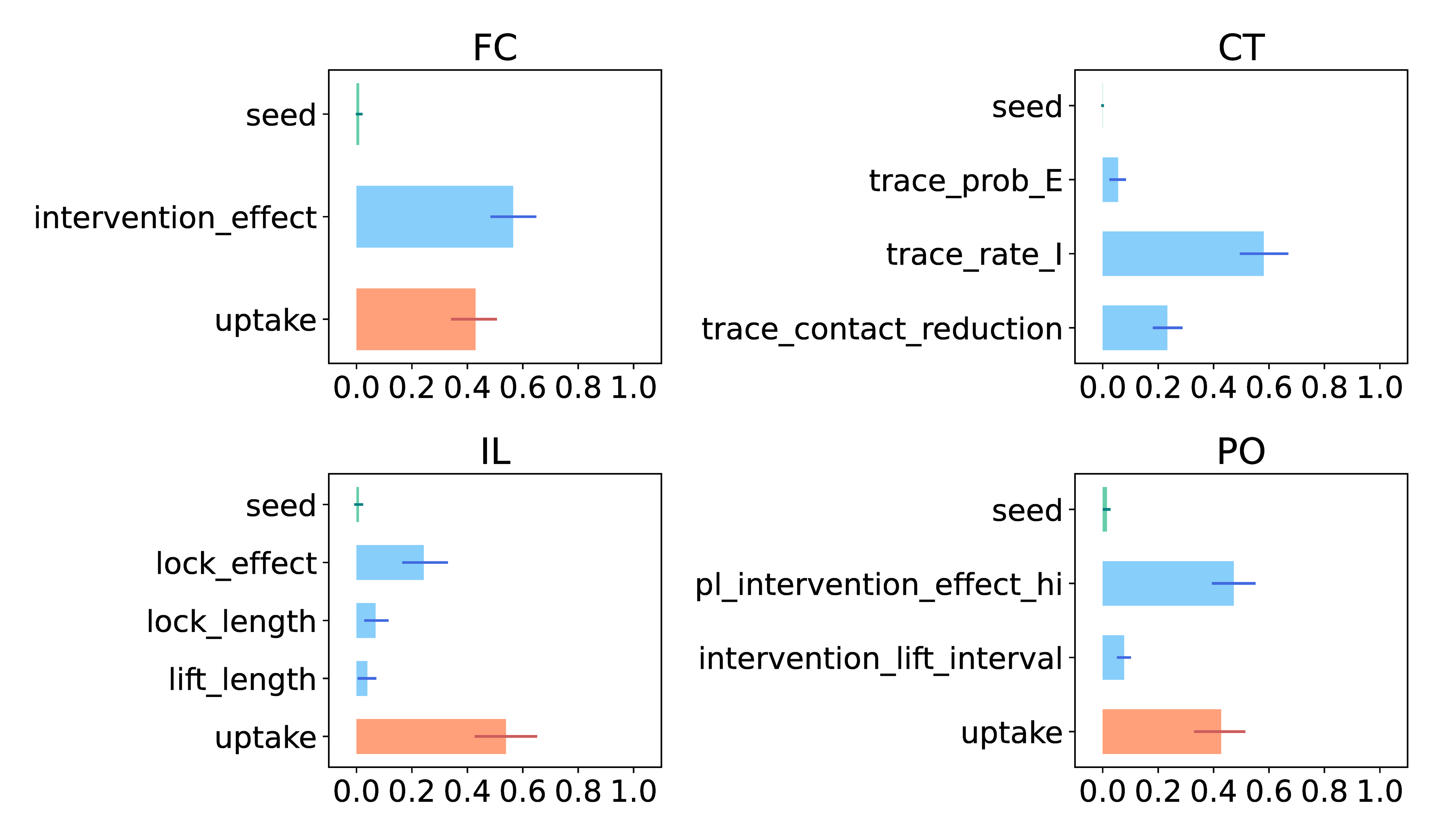

Supplement: S4 Fig — First order Sobol indices of the second QoI (the total number of IC patient-days in excess of IC bed capacity). The length of the bars indicate the mean values, while the thinner lines display the 95% confidence interval. We color in orange the uptake parameter, and in green seed. (TIF) [file pcbi.1009355.s008.tif]

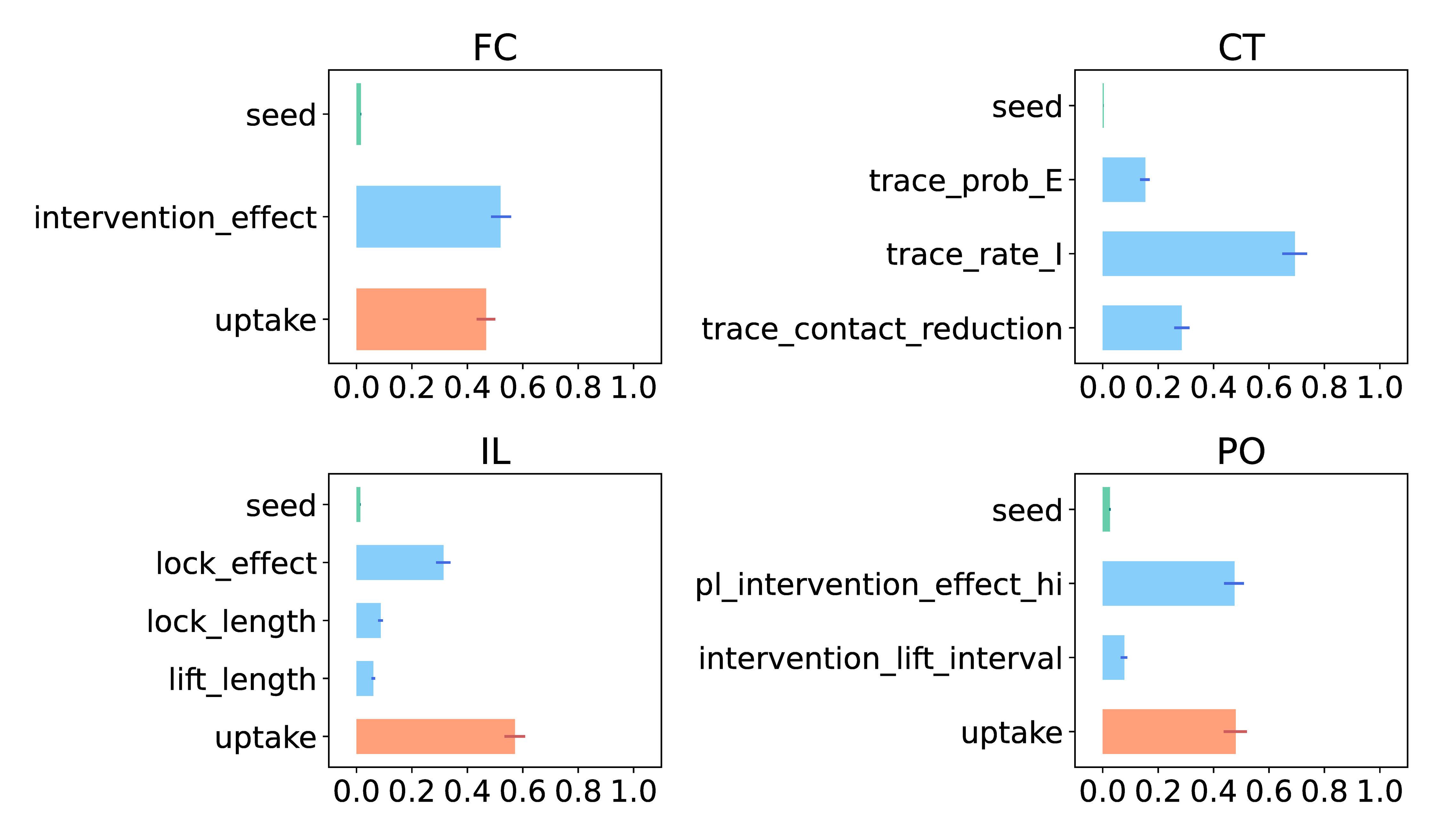

Supplement: S5 Fig — Total Sobol indices of the second QoI (the total number of IC patient-days in excess of IC bed capacity). The length of the bars indicate the mean values, while the thinner lines display the 95% confidence interval. We color in orange the uptake parameter, and in green seed. (TIF) [file pcbi.1009355.s009.tif]
