# Supplementary material for: Uncertainty quantification and sensitivity analysis of COVID-19 exit strategies in an individual-based transmission model
Source: PLoS Comput Biol. 2021 Sep 17;17(9):e1009355. doi: 10.1371/journal.pcbi.1009355 (PMC8480746; doi:10.1371/journal.pcbi.1009355)
Supplement: Attachment — Submitted filename: Response_PLOS_virsim.pdf [file pcbi.1009355.s010.pdf]

Dear Dr. Klinkenberg and Dr. Pitzer,

We would like to thank the editor and the reviewers for their careful reading, constructive criticism and for the opportunity to resubmit. Please find attached our response to the review comments as well as a revised manuscript. Within the manuscript we have marked changes pertaining to Reviewer #1 in red, those pertaining to Reviewer #2 in blue and those pertaining to Reviewer #3 in orange. The adjustments pertaining to the comment from the editor are marked in green.

Best regards,

Federica Gugole (on behalf of the authors)

**Editor comment**
We thank the Editor for their careful reading of the manuscript and the constructive criticism. We have marked adjustments pertaining to this report in green.

*The reviewers were in general enthusiastic about the topic and the quality of writing. I too think that uncertainty analysis in modelling policy advise is important, but I think the manuscript currently does not discuss practical implementation sufficiently. It is said that the model is conceptual, and are an illustration of the UQ and SA approaches, but the results and especially discussion are pretty much focussed on the results of the COVID-19 control scenarios, and less about what the UQ and SA would contribute to the decision making process. I think that should be central: when and why use UQ, and when and why use SA, and how does this improve decisions about the right policy to choose? After all, the manuscript was submitted for the Methods section, and although COVID-19 itself is topical, the strategy comparison in this manuscript is not anymore, so it really should serve as an illustration.*

In the revised manuscript we discuss more in detail the relevance of UQ and SA to the decision making process in the Introduction (page 3 lines 51-67) and in the Discussion (page 17-18, lines 553-568). We also note that practical implementation issues such as computational resources and software are discussed in the last part of the Discussion section (lines 660-688).

**Response to Reviewer #1**

We thank the Reviewer for their careful reading of the manuscript and the constructive criticism. We have marked adjustments pertaining to this report in red.

*This is a very well-written paper and I read it with enthusiasm.*

Thank you for your positive feedback. We are happy to read that you enjoyed our manuscript.

*I had a few suggestions:*

1. *The intermittent lockdown is assumed to go into effect periodically with a prespecified duration of lockdown and then a prespecified duration of opening. I am not sure if this is realistic. If possible, I'd suggest modeling this on/off decision as a function of some epidemic threshold; for example, lockdown when the number of COVID-19 patents in ICU passes a threshold T1 and reopen when the number of COVID-19 patients in ICU goes below a threshold T2. This is more consistent with the way decisions about physical distancing measures have been made and modelled in existing studies.*

The reviewer is right and setting a pre-specified duration for the lockdown and opening periods is not very realistic. Unfortunately a switch based on the number of COVID-19 IC patients (or any other variable for that matter) is not available in the current version of the virsim model. Thus we opted for preset periods of opening/lockdown. This is now more clearly written in the text (page 7-8 lines 277-281).

2. *Please explain briefly why the 'random number seed' is also considered as a parameter (line 346). I am assuming this is to ensure that the Sobol index of the random number seed is negligible since otherwise, it suggests a problem with the random number generator.*

We extended the motivation in the Methods section for considering the random seed as a parameter (page 5 lines 166-169 and page 10 lines 356-361). As can be seen in the newly

added figure S2_Fig in the Supporting information, the virsim model in our setup is quite stochastic, with differences up to 25% in the peak value of the number of prevalent cases in IC due only to variations in the random seed. Nevertheless, the seed has only a small Sobol index (see Fig. 3, S3 Fig, S4 Fig and S5 Fig in the Supporting information), implying that the sum of contributions from the other (non-seed) uncertain parameters to the total variance of the chosen QoIs is much larger than the contribution from the internal stochasticity. There is however no a-priori reason that this is always the case and that Sobol index for the random seed should always be small.

3. *There are other approaches for sensitivity analysis and quantification of uncertainty such as the use of partial-rank correlation: https://www.ncbi.nlm.nih.gov/pmc/articles/PMC3730677/. I think the paper would benefit from a more detailed discussion of alternative methods for SA and UQ.*

We thank the reviewer for signaling us this interesting publication. It is now among our references (Reference #34). We deepened the discussion on the topic in the Discussion section (page 18 lines 601-607). We also added two new references to review papers on available SA techniques and their link to uncertainty analysis (References #31 and #32).

### Response to Reviewer #2

We thank the Reviewer for their careful reading of the manuscript and the constructive criticism. We have marked adjustments pertaining to this report in blue.

*This manuscript provides a well-done and much needed discussion of public-health measures against the COVID-19 pandemic. It is extremely timely and provides well-founded answers to the highly relevant question how effective the various policy measures are.*

Thank you for your positive feedback. We appreciate your support of our work.

*The numerical results can be improved given more computational resources. Major points that should be addressed in a revision are the following.*

- *Regarding the computational model (page 3), it would be convenient for future readers to show the (well-known) SEIR model equations and especially how the geographical stratification works. This could also be done in an appendix, but I believe it would be preferable to show the basic equations and the geographical stratification at the beginning to help the reader to understand how and where the various parameters enter the system of equations. In this regard, it would also be useful to move Table 1 up close to the equations so that the reader can find this important information, i.e., the system of equations and all parameters, in one place at the beginning.*

We included an appendix (S1_Appendix in the Supporting information) showing the model equations and explaining how the geographical stratification works. We refrain from including this in the main text as it is rather lengthy and technical and therefore might discourage readers that are not too keen to know the technical details of the model.

- *On page 3, it is said that "this study is conceptual in nature". Still, it does a very good job at trying to be realistic. A discussion of what is still needed to move from a concept to a realistic treatment would be beneficial in order to help the reader understand any shortcomings if they exist.*

Such discussion is part of the Discussion section (notably, lines 660-688). We added a clear reference to it (page 4 lines 104-106).

- *On pages 6 ff., it is mentioned that Beta and Gamma distributions are assumed for the parameters and some justification is given on page 6. However, would it be possible to provide more specific justifications on these pages where the parameters are discussed?*

We extended the discussion about the distribution choice. In our case the decision was mainly driven by expert knowledge (we note that the authors of this study include the authors of [5], who built the virsim model and hence are experts on the model and its parameters). The extra paragraph on page 6-7 (lines 227-239) is signed as being added in response to Reviewer #3, but addresses your comment as well.

- *In Table 1, references (if available) to justify the choice of the parameter values would be very welcome and add a lot to the discussion.*

This point has also been raised by Reviewer #3 and the adjustments in the text are signed as to be in their reply (page 6-7 lines 227-239 and page 9 lines 326-328). For the policy-related parameters we relied on expert knowledge; for the non-policy-related parameters we relied on a quantification performed by Dablader and Coffeng (2021) and expert knowledge.

- *On page 9, it is mentioned that the geographical stratification in the model adds to the uncertainty in the model via additional parameters. More unknown parameters mean more uncertainties. Therefore the question arises if the additional geographical parameters are indeed advantageous in the sense that they yield an improved model. Does the improvement in the model justify the complications? These questions should be discussed.*

We briefly mentioned a few benefits of the geographical stratification at the beginning of the Computational Model section (page 3-4 lines 88-91). On one hand the geographical stratification adds parameters to the model making it more uncertain; on the other hand it makes it more realistic and allows for heterogeneity in the population and the evaluation of regional measures. This could be useful to evaluate, for instance, how the virus spreads nationwide if different measures are implemented at the regional level (e.g. how the Italian government dealt with the virus during the winter 2020/21 declaring regions 'yellow', 'orange' or 'red' according to the local number of infected). Unfortunately there is always a trade-off between complexity and uncertainty (as well as overfitting risk) when choosing a model. A discussion on the topic has been added to the Discussion section (page 19 lines 637-649).

- *On page 12 it is mentioned that the number M of runs would need to be increased substantially for more accurate estimates of the Sobol indices. Therefore the computational cost (CPU hours) should be discussed a bit more detailed. The discussion in the discussion section comes a bit late and I think it would be good to move some statements about the computational cost up.*

We think that moving some statements about the computational cost up risks giving an incomplete and confusing idea on the topic, while moving the whole related discussion would be premature. We added an explicit reference (page 13 line 465) to the related discussion in the Discussion section .

- *On page 17, the references [7, 8] regarding the use of Bayesian inference to estimate the distributions of the input parameters are very general. Also reference [35] is cited in the*

*context that UQ techniques have (surprisingly) seldom been applied to epidemiological models. An exception I could find is the following work, where such Bayesian calculations were performed already a year ago; the authors might want to consider citing it, also in the context of Table 1.*

*[Leila Taghizadeh, Ahmad Karimi, and Clemens Heitzinger. Uncertainty quantification in epidemiological models for the COVID-19 pandemic. Computers in Biology and Medicine, 125(104011):1--11, 2020.]*

We thank the reviewer for signalling this publication to us; we were not aware of it. It is now included among our references (Reference #35) together with an interesting publication of Gilbert et at (Reference #39) that was also recently brought to our attention.

- *In the discussion on page 18, the computational cost is approximately given. More data would be very useful. How long does one simulation take? Also, is the geographic stratification in the simulations worth it? It has the drawback that it increases computational cost and adds more (uncertain) parameters. Also it seems as if the geographical part of the model was left constant in this work anyway.*

In this work we kept the geographical part of the model fixed. From our point of view it is worthy to have the geographical stratification of the model as it allows assortative mixing in the population (see current S1_Appendix for the technical details) and the evaluation of strategies that include regional measures as, for instance, Phased Opening.  A discussion on the worth of the geographical stratification has been added (page 19 lines 637-649).

We included the computational cost of one simulation of the virsim model (page 20 lines 667-670). However it is difficult to give an accurate estimate of the exact computational cost as it depends on the model itself, the available infrastructure and software. The supercomputer at the Poznan Supercomputing and Networking Center has older and newer CPUs; the exact computational cost of our campaigns can vary due to which nodes were available at the moment and queueing time. Also, new releases of the software (FabSim, EasyVVUQ and QCG-PJ) have come out since we carried out our analysis; this would likely lead to reduced computational costs for the simulations.

### Response to Reviewer #3

We thank the Reviewer for their careful reading of the manuscript and the constructive criticism. We have marked adjustments pertaining to this report in orange.

*Gugole et al. present a thorough uncertainty quantification and sensitivity analysis of an agent-based model of SARS-CoV-2 transmission. The model is based on the Netherlands, but the paper is really focused on describing these methods carefully and discussing the interpretation of their output, rather than attempting to make realistic projections. They consider four strategies for reopening, and find that, for each strategy, 2-3 key parameters largely determine whether ICU capacity will remain manageable, and that these key parameters are always policy-related parameters.*

*I very much enjoyed reading the paper. It is well written, explains the methods clearly, and would be a very useful read for an infectious disease modeler (or any modeler) who wishes to learn and employ UQ and SA in their work.*

Thank you for your positive feedback. We are happy to read that you enjoyed our manuscript.

*I only have a few minor comments, detailed below:*

*Methods and model*

- *Line 73: you could refer to S1 Fig here, as the reader may wish to visualize the distributions of these parameters.*

We added the reference to S1 Fig (page 4 line 93). Thank you for the suggestion.

- *What sampling method did you use to sample from the parameters distributions in the UQ?*

We used the Monte Carlo sampler. More details are given in the UQ and SA computational framework (page 10 lines 383-390).

- *Line 208: write the date to which you reconstruct rather than 'present time'*

We specified the date to which we reconstruct (page 7 lines 245-246).

- *Please give references for the distributions of the non-policy related parameter ranges you use, e.g. R0, infectiousness duration etc. If possible, it would be nice to see references supporting your choice of distributions for the policy related parameters, too. I think at the moment, the rather hand-wavy justification for these distributions may be the weakest part of the manuscript, although I appreciate that most of these parameters may not have much literature to support your choices.*

The distributions for the policy-related parameters have been determined by expert knowledge (we note that the authors of this study include the authors of [5], who built the virsim model and hence are experts on the model and its parameters). Finding literature supporting our choices is very hard, if not impossible. The meaning of most of these parameters is model specific therefore even if other studies might have had distributions for this type of parameters, very likely they would not be transferable to our model.

Regarding the non-policy-related parameters we selected distributions whose mean is (almost) the value obtained in a previous quantification of the model performed by Dablader & Coffeng (2021) and explored the model outcome for parameter values reasonably close to the mean. Here too we used expert knowledge to determine the overall shape of the distributions.

This is more clearly stated in the revised version of our manuscript (page 6-7 lines 227-239, page 8 lines 326-328 and page 10 lines 373-374).

- *Line 337: Could you explain your choice of 1000 simulations for the UQ and the choice of M=2000 for the SA.*

We updated the UQ analysis using 2000 simulations, making it consistent with the number of simulations for SA.

- *On this subject, it's not completely clear whether you do 1000 simulations per parameter set or 1000 overall? I'm assuming the former, but probably best to make this clear around line 338*

We did 1000 (now 2000) simulations in total for the UQ analysis. We used MC sampling, implying that for each simulation, the full vector of uncertain parameters is sampled anew

(randomly, independently). It is more clearly written in the current version of the manuscript (page 10 lines 386-387).

For the SA we did M=2000 simulations per parameter because of how the Saltelli algorithm is designed, but for the same reason not all the simulations in the SA analysis are independent from each other. Therefore the SA analysis contains many more simulations than the UQ analysis.

- *In Table 1, could you indicate in some way which type of parameter each is (i.e. policy-related or other), and if policy-related, which strategies it pertains to?*

We added a column to Table 1 indicating to which strategy each parameter pertains or if it is a non-policy-related parameter (see page 9).

*Results*

- *Could you show a supplementary figure of one reopening scenario, similar to Figure 1, but where each line uses the same parameter set? Perhaps with more than four lines – something like a spaghetti plot would work well. This would help the reader to get a sense of how stochastic the model is.*

We included such a plot in the supplementary material (S2 Fig in the Supporting information).

- *Line 407: could you include the SA for the other QoI as a supplementary figure?*

We included the plots of the first order and total Sobol indices with respect to the second QoI (see S4 Fig and S5 Fig in the Supporting information).

- *Figure 3: Could you show the higher order terms on this plot? Judging from the x-axis they must be pretty small, but it would still be nice to see it.*

We did not estimate the higher order terms as this would require many more simulations thus greatly increasing the computational cost (page 14, lines 492-493). A rough estimate can be obtained by comparing the first order indices (Fig 3) to the total indices (S3_Fig).

- *Line 443: Could you show the second order interaction terms in a supplementary table or figure (e.g. a heat-map)?*

The second order interactions can be seen in the scatter plots used to determine the safe operating space. For instance in case of Flattening the Curve (Fig 4) it can be seen that there is a clear color gradient (indicating the direction of increasing/decreasing numbers of IC patients) orthogonal to a sloped line linking the uptake by the population to the relative level of transmission due to intervention. This is mentioned now also in the manuscript (page 14 lines 493-494).

*Discussion*

- *Line 512: I'm not sure "most effective" is the correct term here, because as you say, it depends on the parameters. Perhaps it would be better to say "has the most potential to avoid exceeding ICU capacity", or something like that?*

We rephrased that sentence (page 18 lines 579-580). Thank you for the suggestion.
